# Cardiogenic Shock Among Patients with and without Acute Myocardial Infarction in a Latin American Country: A Single-Institution Study

**DOI:** 10.5334/gh.988

**Published:** 2021-11-30

**Authors:** Héctor González-Pacheco, Daniel Manzur-Sandoval, Rodrigo Gopar-Nieto, Amada Álvarez-Sangabriel, Carlos Martínez-Sánchez, Guering Eid-Lidt, Alfredo Altamirano-Castillo, Salvador Mendoza-García, José Luis Briseño-Cruz, Francisco Azar-Manzur, Diego Araiza-Garaygordobil, Daniel Sierra-Lara, Gian Manuel Jiménez-Rodríguez, Emmanuel Adrián Lazcano-Díaz, Francisco Baranda-Tovar, Jessrel Sharon Valencia-Älvarez, Miguel Alejandro Cutz-Ijchajchal, Jose Carlos Penagos-Cordon, Paola Morejon-Barragán, Alexandra Arias-Mendoza

**Affiliations:** 1 Coronary Care Unit, National Institute of Cardiology in Mexico City, MX; 2 Heart Failure Clinic and Transplantation, National Institute of Cardiology in Mexico City, MX; 3 Department of Interventional Cardiology, National Institute of Cardiology in Mexico City, MX; 4 Cardiovascular Critical Care Unit, National Institute of Cardiology in Mexico City, MX; 5 Cardiovascular Critical Care Unit, Instituto Argentino de diagnóstico y tratamiento, Buenos Aires, AR

**Keywords:** Cardiogenic shock, Cardiogenic shock without AMI, Acute myocardial infarction, Latin America, Middle-income countries, Acute Heart Failure

## Abstract

**Background:**

Latin America has limited information about the full spectrum cardiogenic shock (CS) and its hospital outcome. This study sought to examine the temporal trends, clinical features and outcomes of patients with CS in a coronary care unit of single Mexican institution.

**Methods:**

This was a retrospective study of consecutive patients hospitalized with CS in a Mexican teaching hospital between 2006–2019. Patients were classified according to the presence or absence of acute myocardial infarction (AMI).

**Results:**

Of 22,747 admissions, 833 (3.7%) exhibited CS. Among patients with AMI (n = 12,438), 5% had AMI–CS, and in patients without AMI (n = 10,309), 2.3% developed CS (non-AMI–CS). Their median age was 63 years and 70.5% were men. Cardiovascular risk factors were more frequent among the AMI–CS group, whereas a history of heart failure was greater in non-AMI–CS patients (70.1%). In AMI-CS patients, the median delay time was 17.2 hours from the onset of AMI symptoms to hospital admission. Overall, the median left ventricular ejection fraction (LVEF) was 30%. Patients with CS at admission showed end-organ dysfunction, evidenced by lactic acidosis, renal impairment, and elevated liver transaminases. Of the 620 AMI–CS patients, the main cause was left ventricular dysfunction in 71.3%, mechanical complications in 15.2% and right ventricular infarction in 13.5%. Among the 213 non-AMI–CS patients, valvular heart disease (49.3%) and cardiomyopathies (42.3%) were the most frequent etiologies. In-hospital all-cause mortality rates were 69.7% and 72.3% in the AMI–CS and non-AMI–CS groups, respectively. Among AMI–CS patients, renal dysfunction, diabetes, older age, depressed LVEF, absence of revascularization and the use of mechanical ventilation were independent predictors of in-hospital mortality. However, in the non-AMI–CS group, only low LVEF and high lactate levels proved significant.

**Conclusions:**

This study demonstrates differences in the epidemiology of CS compared to high-income countries; the high mortality reflects critically ill patients and the lack of contemporary effective therapies in the population studied.

## Introduction

Cardiogenic shock syndrome (CS) is the most severe form of cardiac decompensation with end-organ hypoperfusion, clinical decompensation with multisystem organ failure, and subsequent death if a reversible cause is not identified and managed. The most common cause of CS is acute myocardial infarction (AMI), mainly caused by left ventricular dysfunction; less frequently, mechanical complications are causative but are not related to infarct size [1,2]. Nevertheless, the etiological spectrum is broad and many other nonischemic etiologies, such as end-stage cardiomyopathy, myocardial contusion, myocarditis, cardiac hypertrophy, valvular heart disease and pericardial disease, can also lead to CS [3,4].

The primary data available for patients with CS come from several large-scale registries of ST-segment-elevation myocardial infarction (STEMI), from high-income countries [5,6,7]. Despite the income level and despite treatment advances made in the last decade, mortality rates continue to be as high as 40–60%, including early myocardial revascularization in patients with AMI [8,9,10]. The CS is an area of burgeoning interest not only in North America and western Europe but also in low- and middle-income countries, reflecting the demographic changes and the epidemiological transition to cardiovascular diseases occurring in the latter countries. It has been documented that heart failure patients present considerable differences in outcome between high-income, low- and middle-income countries [11]. There is little published data about prevalence, ischemic and nonischemic etiologies, management and results of unselected patients with CS in low- and middle-income countries. This there is a need for better clinical understanding of the heterogeneous causes and presentation of CS with the aim of tailoring therapies to improve patient outcomes [12].

Because information on patients with CS in low- and middle-income countries is scarce, we conducted a retrospective analysis to determine the prevalence, temporal trends, baseline characteristics, etiologies, management and in-hospital outcomes of consecutive patients with CS admitted to the coronary care unit (CCU) of a contemporary teaching hospital in Mexico City.

## Materials and Methods

This was a retrospective, single-center cohort study using data from the CCU database of the National Institute of Cardiology in Mexico City. We analyzed data from all patients admitted consecutively from January 2006 to December 2019. We gathered basal demographic data, clinical characteristics, and information related to the patient’s clinical evolution during their stay at the CCU. To establish a clinical diagnosis of CS, we used the definition from the IABP-SHOCK II study with clinical criteria of systolic blood pressure <90 mmHg for ≥30 min or the need for catecholamines to maintain systolic blood pressure >90 mmHg [8], clinical pulmonary congestion, and organ hypoperfusion with any of the following symptoms: cold extremities; confusion or altered mental status; oliguria, or; blood lactate >2.0 mmol/L. We identified patients with CS and classified them according to etiology. Cardiogenic shock associated with AMI, including STEMI or non-ST-segment elevation myocardial Infarction (NSTEMI), was defined as CS caused by AMI (AMI–CS). Cardiogenic shock with a nonischemic etiology was defined as non-AMI–CS.

The diagnosis of AMI was based on clinical characteristics, electrocardiographic changes, and blood levels of biochemical markers of cardiac necrosis (creatinine kinase isoenzymes, creatinine phosphokinase or troponin I), and it was classified as STEMI or NSTEMI according to the American College of Cardiology criteria [13].

In patients with non-AMI–CS, the following primary underlying etiologies were documented [1]: valvular heart disease of organic etiology including endocarditis and valvular prosthesis dysfunction [2]; cardiomyopathies, including idiopathic dilated, hypertrophic and hypertensive types, peripartum, left ventricular noncompaction, Chagas disease cardiomyopathy, restrictive cardiomyopathy, chronic ischemic cardiomyopathy and myocarditis [3]; lung diseases such as pulmonary embolism, pre-existing disease, or arterial pulmonary hypertension [4]; pericardial disease [5]; intracardiac tumors [6]; adult congenital heart disease, and [7]; acute aortic syndromes. Patients with CS were further classified according to whether they presented with CS at the time of admission or developed CS during CCU stay (late CS).

The primary outcome of the study was in-hospital all-cause mortality. In-hospital death was divided into cardiovascular (CV) and noncardiovascular CV death. CV death was defined as death caused by refractory CS, sudden cardiac death or death caused by stroke. Non-CV death was defined as death caused by infections, renal failure, liver failure or multiple organ failure.

## Statistical analyses

For descriptive analyses, continuous variables were tested and confirmed to be nonnormally distributed as determined by Kolmogorov–Smirnov tests and are presented as medians and 25th and 75th percentiles (interquartile ranges, IQRs) The Mann–Whitney nonparametric *U* test was used for comparisons between two groups. Categorical variables are expressed as proportions and percentages, and the differences were assessed using the chi-squared test or Fisher’s exact probability test, as appropriate. The study outcomes were defined as all-cause hospital mortality. An age- and gender-adjusted Cox proportional hazards regression model was used to estimate the association between each CS group and their in-hospital risk of death, compared with patients without CS. Hazard ratios (HRs) with 95% confidence intervals (CIs) were calculated. Univariate and multivariate Cox’s proportional hazards regression models with backward selection were generated separately for the AMI–CS and non-AMI–CS groups and were used to identify significant predictors of the end point of in-hospital all-cause mortality. Candidate covariates included in the multivariate analysis were selected from clinical variables at the time of admission (demographic variables, cardiovascular risk factors, medical history, clinical features on presentation and in-hospital cardiac procedures) that were associated with mortality in a univariate analysis with P < 0.05.

All tests were two-sided, and P ≤ 0.05 was considered to be statistically significant. IBM SPSS Statistics for Windows was used for all analyses (v. 23; IBM Corp., Armonk, NY, USA).

## Results

**Figure 1:**
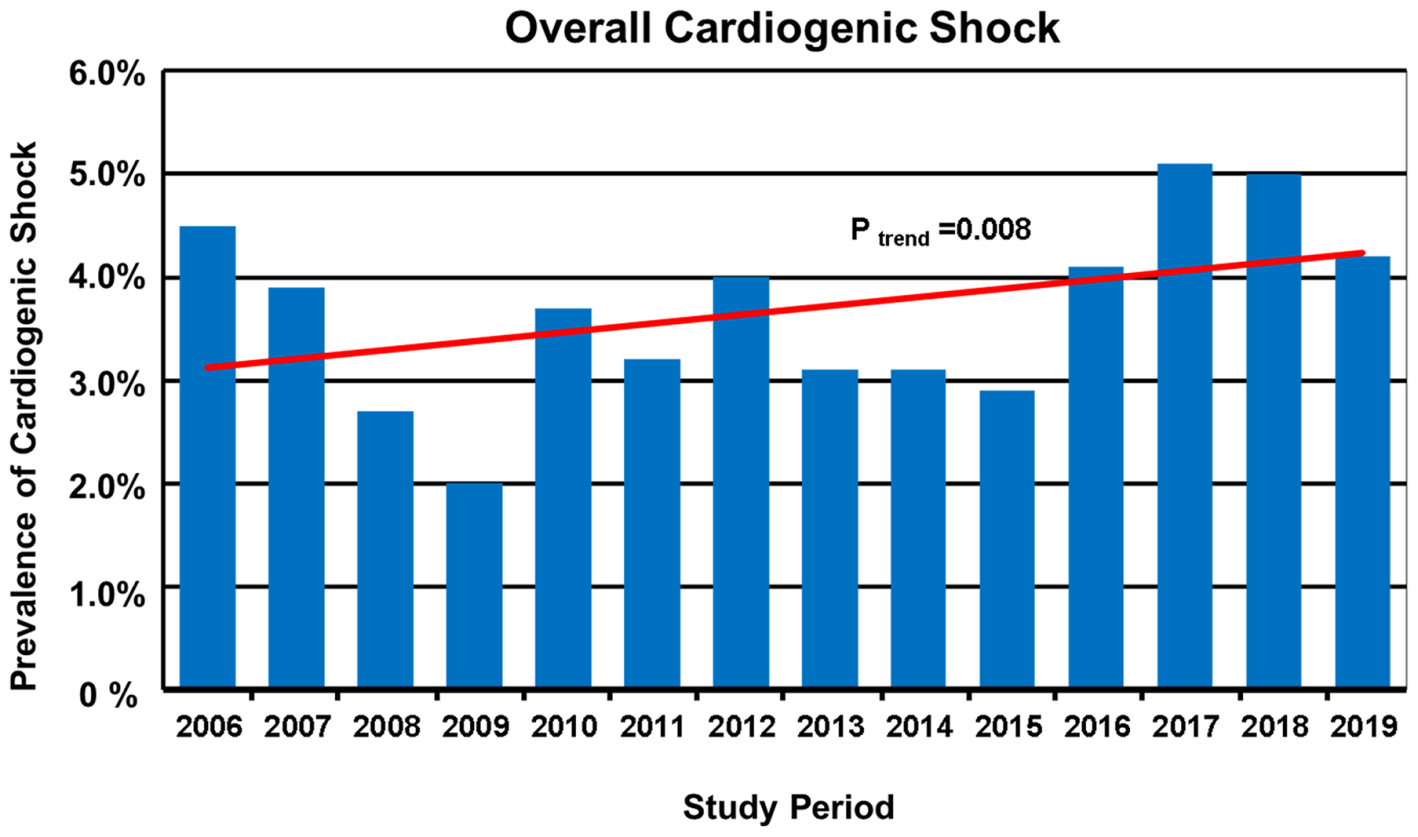
Rates of patients with overall cardiogenic shock (CS) hospitalized by study year between 2006 and 2019 (total n = 22,747).

**Figure 2:**
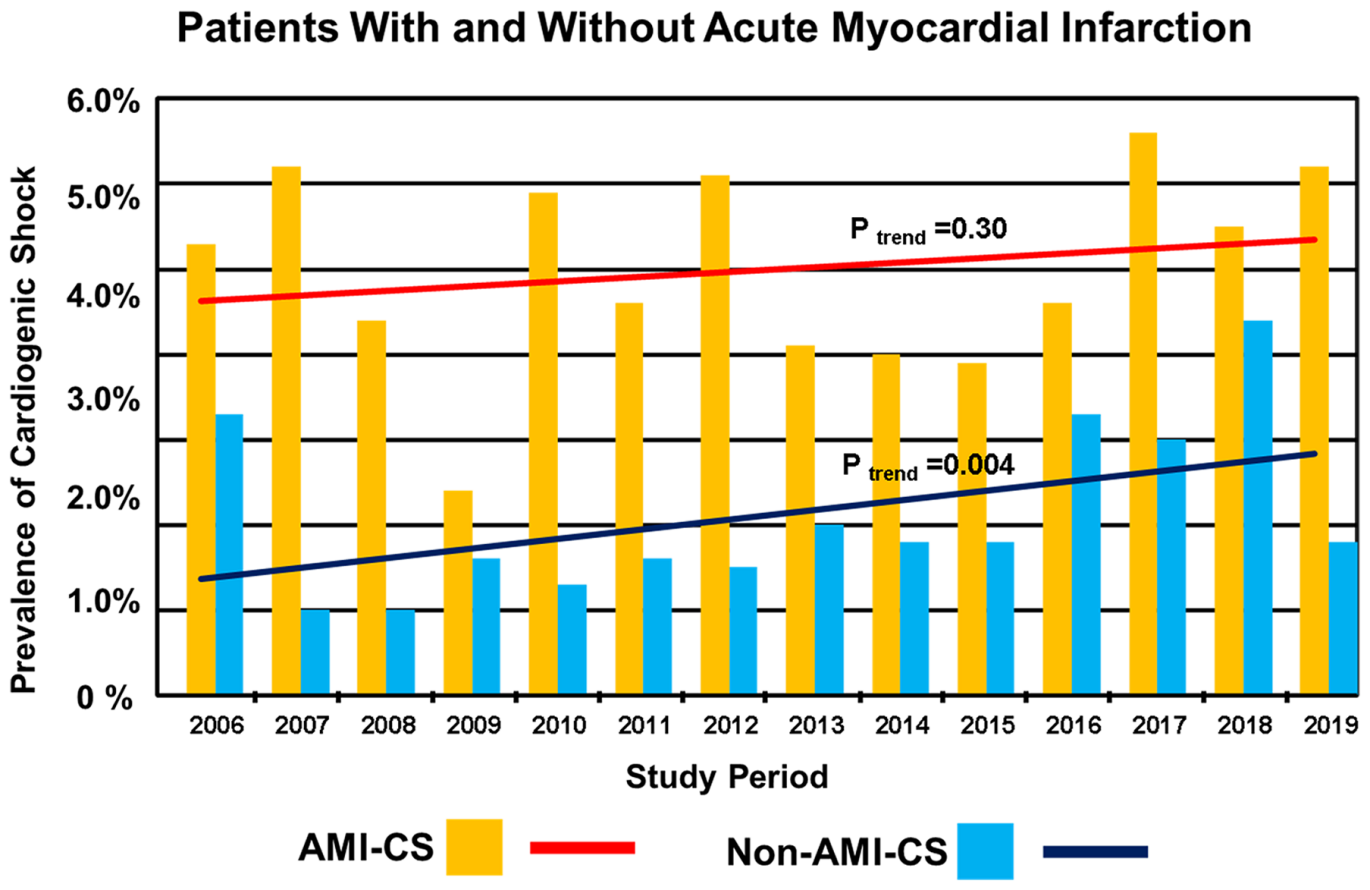
Rates of patients with cardiogenic shock (CS) associated with acute myocardial infarction (AMI–CS) in patients hospitalized with AMI (n = 12,438) and CS associated with nonischemic etiologies (non-AMI–CS) in patients hospitalized by others causes not related to acute myocardial infarction (n = 10,309) by study year between 2006 and 2019.

During the analyzed period from January 2006 to December 2019, 22,747 patients were admitted consecutively to the CCU. Among these, CS was documented in 833 (3.7%). In the period analyzed, the overall rate of CS had statistically significant upward trend over time (P _trend_ = 0.008; Figure 1). Among the 12,438 patients hospitalized with confirmed AMI during the years of this study, 620 (5.0%) developed CS. On the other hand, in 10,309 patients without AMI, 213 (2.1%) developed CS (Figure 2). However, in the course of the 14 years analyzed, there were no significant changes in the trend in rates over time for those with AMI-CS (P _trend_ = 0.30) but there was a slight but statistically significant upward trend in rates over time for those whit non-AMI-CS (P _trend_ = 0.004; Figure 2).

The baseline characteristics of the study population are shown in Table 1. Overall, the median age was 63 years and most of them were men (70.5%). Significant differences were observed between the AMI–CS and non-AMI–CS groups. Patients with AMI–CS were older (median 64 years [IQR, 55–72 years] versus median 57 years [IQR, 45–69 years], P < 0.0001) and significantly more likely to have multiple risk factors, such as a history of diabetes, hypertension, dyslipidemia, smoking, myocardial infarction, percutaneous coronary intervention or coronary bypass surgery. In contrast, women were more prevalent in the non-AMI–CS group (47.4% vs. 23.4%; P < 0.0001) and were more likely to have a history of heart failure, stroke or atrial fibrillation. There was a history of heart valve surgery in 20.7% of these patients.

**Table 1:** Baseline Characteristics of Patients With AMI-CS and non-AMI-CS.

	Overall (n = 833)	AMI-CS (n = 620)	Non-AMI-CS (n = 213)	*P*- Value

**Age median, (IQR) (years)**	63 (53–71)	64 (55–72)	57 (45–69)	<0.0001
**Men, n (%)**	587 (70.5)	475 (76.6)	112 (52.6)	<0.0001
**Body mass index median, (IQR) (kg/m^2^)**	26.3 (23.8–29.3)	26.6 (24.2–29.4)	25.1 (22.2–27.6)	<0.0001
**Medical History**				
**Current smoking, n (%)**	205 (24.6)	188 (30.3)	17 (8.0)	<0.0001
**Hypertension, n (%)**	420 (50.4)	346 (55.8)	74 (34.7)	<0.0001
**Dyslipidemia, n (%)**	231 (27.7)	198 (31.9)	33 (15.5)	<0.0001
**Diabetes, n (%)**	371 (44.5)	319 (51.5)	52 (24.4)	<0.0001
**Previous MI, n (%)**	159 (19.1)	127 (20.5)	32 (15.0)	0.08
**Previous CABG, n (%)**	21 (2.5)	15 (2.4)	6 (2.8)	0.74
**Previous PCI, n (%)**	73 (8.8)	57 (9.2)	16 (7.5)	0.45
**Previous heart failure, n (%)**	229 (27.5)	69 (11.1)	160 (75.1)	<0.0001
**Previous stroke, n (%)**	49 (5.9)	24 (3.9)	25 (11.7)	<0.0001
**Previous atrial fibrillation, n (%)**	88 (10.6)	16 (2.6)	72 (33.8)	<0.0001
**Previous valvular surgery, n (%)**	47 (5.6)	3 (0.5)	44 (20.7)	<0.0001

AMI-CS, Cardiogenic Shock caused by Acute myocardial infarction; non-AMI-CS, Cardiogenic shock of non-ischemic etiology; MI, myocardial infarction; CABG, coronary artery bypass grafting; PCI, percutaneous coronary intervention.

In all, 451 (54.1%) patients developed CS at admission and 382 (45.9%) developed CS in hospital. In patients with AMI-CS at admission, the median time from symptom onset to arrival was 17.2 (IQR, 6.2–47.2) hours. Only 121 (41.4%) patients presented within the first 12 hours of the onset of symptoms, 51 (17.5%) patients were admitted within 12 to 24 hours and 120 (41.1%) patients 24 hours after the symptoms onset. Tables 2 and 3 outlines hemodynamic and laboratory values grouped by presence of CS at the time of admission or developed CS during CCU stay. In both scenarios, the left ventricular ejection fraction (LVEF) was low (median 30% vs 30%, respectively) and patients with non-AMI–CS had lower blood pressure, lower glomerular filtration rates, and higher levels of the N-terminal prohormone of brain natriuretic peptide compared with patients in the AMI–CS group. By comparison, patients with AMI–CS had higher levels of glycemia and high sensitivity C-reactive protein. Interestingly, laboratory values demonstrated significant end-organ dysfunction in patients with CS at admission, as evidenced by lactic acidosis, renal impairment, and elevated liver transaminases. These biochemical derangements were most pronounced in patients with AMI-CS (Table 2).

**Table 2:** Cardiogenic Shock at admission: Clinical features, laboratory data and echocardiographic findings at hospital admission of Patients With AMI-CS and non-AMI-CS.

	Overall (n = 451)	AMI-CS (n = 292)	Non-AMI-CS (n = 159)	*P*- Value

**Heart rate median, (IQR) (beats/min)**	100 (65–113)	100 (56–110)	99 (70–120)	0.11
**Systolic blood pressure median, (IQR) (mmHg)**	80 (70–85)	80 (70–90)	78 (67–80)	<0.0001
**Diastolic blood pressure median, (IQR) (mmHg)**	48 (40–53)	50 (40–60)	40 (34–50)	<0.0001
**Mean blood pressure median, (IQR) (mmHg)**	57 (50–63)	60 (50–67)	53 (47–60)	<0.0001
**LVEF median, (IQR) (%)**	30 (21–40)	30 (21–40)	30 (21–50)	0.15
**Haemoglobin median, (IQR) (g/L)**	13.9 (11.9–15.7)	14.0 (12.4–16.0)	13.2 (11.0–15.1)	0.002
**Blood glucose level, median, (IQR) (mg/dL)**	177 (116–273)	212 (149–301)	120 (89–177)	<0.0001
**Hs-CRP, median (IQR) (mg/L)**	58.9 (20.5–144.2)	68 (27.9–150.0)	47 (17.0–118.0)	0.05
**Alanine aminotransferase, median, (IQR), (U/L)**	88 (40–408)	105 (52–432)	66 (24–353)	0.001
**Aspartate aminotransferase, median, (IQR), (U/L)**	181 (57–649)	273 (83–698)	79 (46–329)	<0.0001
**Albumin, median (IQR) (g/dL)**	3.2 (2.8–3.5)	3.2 (2.8–3.5)	3.2 (2.6–3.6)	0.55
Renal dysfunction* median, (IQR) (ml/min)	36.2 (24.3–56.6)	40.2 (26.5–59.6)	34.5 (20.0–46.7)	0.002
**Blood Lactate, median (IQR) (mmol/L)**	4.6 (2.8–37.9)	4.5 (2.5–7.8)	4.8 (3.0–8.4)	0.06
**Arterial pH, median (IQR)**	7.30 (7.20–7.38)	7.29 (7.20–7.39)	7.31 (7.20–7.37)	0.81
**NT-proBNP median, (IQR) (ng/L)**	14,375 (5,126–25,000)	8,680 (3,360–19,687)	22,826 (11,121–8,858)	<0.0001

* Creatinine depuration ≤60 mL/min at the time of admission ((according to the Cockroft-Gault formula); LVEF, left ventricular ejection fraction; hs-CRP, high-sensitivity C-reactive protein; NT-proBNP, N-terminal prohormone of brain natriuretic peptide.

**Table 3:** Cardiogenic shock developed after admission: Clinical features, laboratory data and echocardiographic findings at hospital admission of Patients With AMI-CS and non-AMI-CS.

	Overall (n = 382)	AMI-CS (n = 328)	Non-AMI-CS (n = 54)	*P*- Value

**Heart rate median, (IQR) (beats/min)**	91 (75–105)	90 (75–114)	100 (86–108)	0.01
**Systolic blood pressure median, (IQR) (mmHg)**	110 (100–130)	112 (100–130)	101 (90–116)	0.001
**Distolic blood pressure median, (IQR) (mmHg)**	70 (60–80)	70 (60–80)	63 (60–71)	<0.0001
**Mean blood pressure median, (IQR) (mmHg)**	85 (74–97)	87 (77–97)	77 (70–87)	<0.0001
**LVEF median, (IQR) (%)**	33 (25–40)	34 (25–40)	30 (22–53)	0.90
**Haemoglobin median, (IQR) (g/L)**	14.3 (12.6–16.0)	14.4 (13.1–16.0)	12.9 (11.2–15.3)	<0.0001
**Blood glucose level, median, (IQR) (mg/dL)**	169 (128–258)	178 (136–278)	126 (103–149)	<0.0001
**Hs-CRP, median (IQR) (mg/L)**	42.3 (13.0–114.0)	43.1 (13.0–112.0)	38.7 (15.4–122.2)	0.91
**Alanine aminotransferase, median, (IQR), (U/L)**	65 (34–139)	72 (39–143)	34 (19–93)	<0.0001
**Aspartate aminotransferase, median, (IQR), (U/L)**	106 (44–338)	136 (50–383)	44 (28–92)	<0.0001
**Albumin, median (IQR) (g/dL)**	3.3 (3.0–3.7)	3.3 (3.0–3.7)	3.2 (2.9–3.6)	0.07
Renal dysfunction* median, (IQR) (ml/min)	57.5 (38.4–84.6)	59.1 (39.0–86.4)	49.4 (26.9–69.6)	0.01
**Blood Lactate, median (IQR) (mmol/L)**	2.0 (1.4–3.2)	2.1 (1.4–3.3)	1.8 (1.3–3.0)	0.10
**Arterial pH, median (IQR)**	7.40 (7.34–7.45)	7.40 (7.33–7.45)	7.44 (7.37–7.49)	0.01
**NT-proBNP median, (IQR) (ng/L)**	6,278 (2,052–17,475)	5,387 (1,578–14,908)	16,905 (9,387–25,000)	<0.0001

* Creatinine depuration ≤60 mL/min at the time of admission (according to the Cockroft-Gault formula); LVEF, left ventricular ejection fraction; hs-CRP, high-sensitivity C-reactive protein; NT-proBNP, N-terminal prohormone of brain natriuretic peptide.

**Figure 3:**
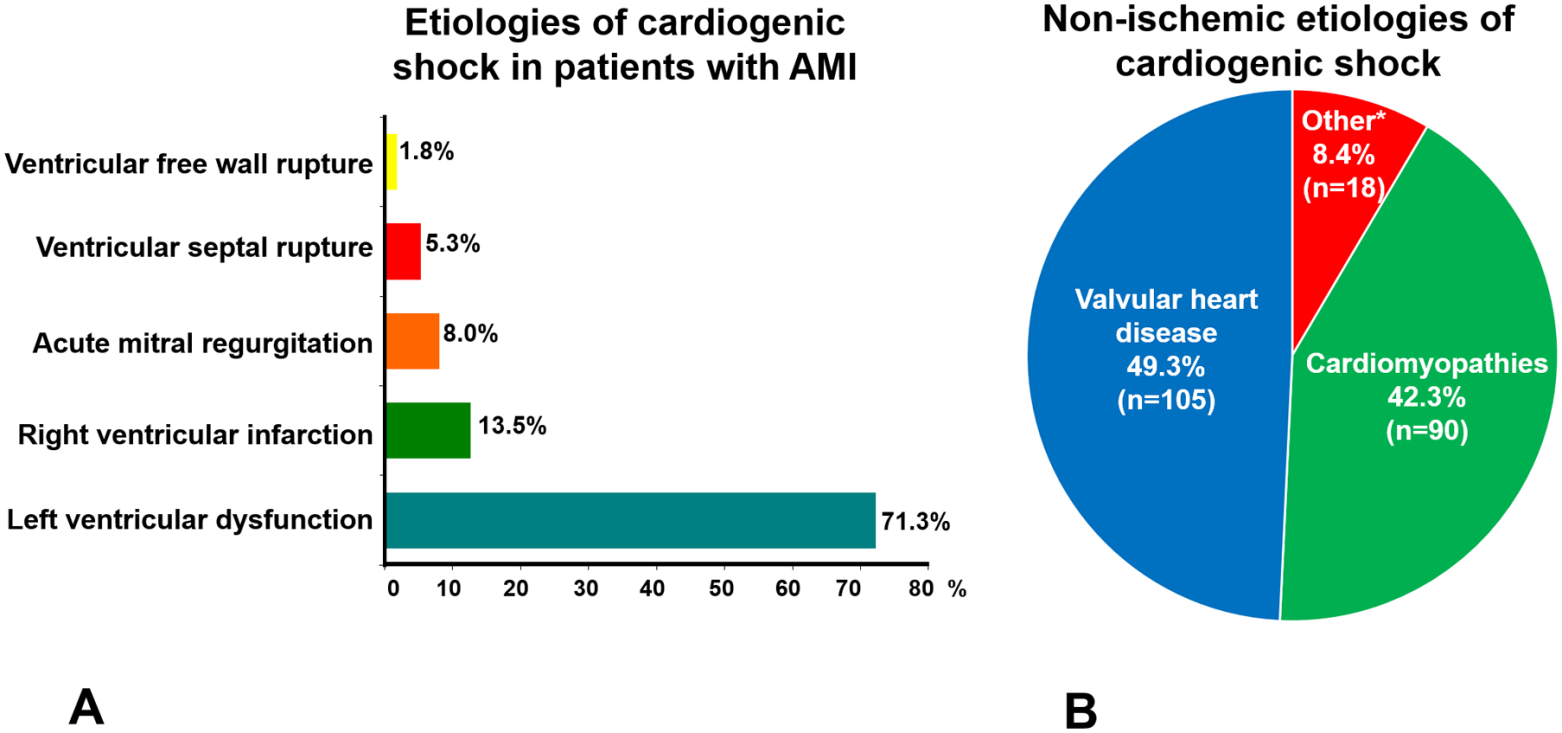
The frequencies of major cardiogenic shock etiologies are shown. **A.** Etiologies of cardiogenic shock in 620 patients with acute myocardial infarction (AMI). **B.** Etiologies of cardiogenic shock (CS) not associated with acute myocardial infarction (AMI) in 213 patients. Cardiomyopathies included idiopathic dilated cardiomyopathies (n = 42), chronic ischemic cardiomyopathy (n = 28), hypertension (n = 4), cardiac hypertrophy (n = 1), peripartum cardiomyopathy (n = 1), left ventricular noncompaction (n = 2), Chagas disease cardiomyopathy (n = 6), restrictive cardiomyopathy (n = 2) and myocarditis (n = 4). * These grouped as ‘other’ included lung disease (n = 10), adult congenital heart disease (n = 5) pericardial disease (n = 1) intracardiac tumor (n = 1), and acute aortic syndrome (n = 1).

***Etiologies of CS:*** Of all patients with AMI–CS, the rates of CS were significantly higher in patients with STEMI (n = 506, 81.6%) compared with those with NSTEMI (n = 114, 18.4%; P < 0.0001). The main cause was left ventricular dysfunction in 71.3% (n = 442), mechanical complications in 15.2% (acute mitral regurgitation in 50 patients, ventricular septal rupture in 33, and ventricular free wall rupture/tamponade in 11). In 84 patients (13.5%), right ventricular infarction was the cause (Figure 3-A).

Among the 213 patients with non-AMI–CS, the most frequent underlying etiologies were valvular heart disease at 49.3% (n = 105) and cardiomyopathies 42.3% (n = 90). In the remaining 18 patients, we found a miscellaneous etiology lung disease (n = 10), adult congenital heart disease (n = 5) pericardial disease (n = 1) intracardiac tumor (n = 1), and acute aortic syndrome (n = 1). (Figure 3-B).

***In-hospital management:*** The median hospital stay was 6 days (IQR, 2–16) for patients with AMI-CS versus 5 days (IQR, 2–13) for patients with non-AMI-CS (P = 0.23). Overall, vasopressors were given to 95.0% of patients (norepinephrine 94.2%, vasopressin 61.8%, or both 61.1%) and inotropic agents to 75.6% (dobutamine 68.4%, levosimendan in 16.0% and dopamine in 12.8%), particularly those with AMI–CS (Table 4). In the whole cohort, other important initial nonpharmacological measures were mechanical ventilation in 67.7%, intra-aortic balloon pump placement in 36.3%, pulmonary artery catheter insertion in 22.0% and renal replacement therapy in 11.8%. Mechanical ventilation, intra-aortic balloon pumps and pulmonary artery catheters were used most frequently in patients with AMI–CS. Among patients with AMI–CS, coronary angiography and percutaneous coronary intervention (PCI) were undertaken in 78.7% and 63.5%, respectively. However, of the 506 patients with STEMI, only 175 (34.6%) received reperfusion therapy with primary PCI (Table 4).

**Table 4:** In-hospital Management and Procedures in Patients With AMI-CS and non-AMI-CS.

	Overall (n = 833)	AMI-CS (n = 620)	Non-AMI-CS (n = 213)	*P*- Value

**Inotropes any, n (%)**	630 (75.6)	501 (80.8)	129 (60.6)	<0.0001
**Dobutamine, n (%)**	570 (68.4)	458 (73.9)	112 (52.6)	<0.0001
**Levosimendan, n (%)**	133 (16.0)	110 (17.7)	23 (10.8)	0.01
**Dopamine, n (%)**	107 (12.8)	79 (12.7)	28 (13.1)	0.87
**Vasopressors any, n (%)**	791 (95.0)	578 (93.2)	213 (100)	<0.0001
**Norepinephrine, n (%)**	785 (94.2)	572 (92.3)	213 (100)	<0.0001
**Vasopressin, n (%)**	515 (61.8)	385 (62.1)	130 (61.0)	0.78
**Both Vasopressors, n (%)**	509 (61.1)	379 (61.1)	130 (61.0)	0.98
**IABP, n (%)**	302 (36.3)	294 (47.4)	8 (3.0)	<0.0001
**Mechanical ventilation, n (%)**	563 (67.6)	436 (70.3)	127 (59.6)	0.004
**Pulmonary artery catheter, n (%)**	183 (22.0)	171 (27.6)	12 (5.6)	<0.0001
**Renal replacement therapy, n (%)**	98 (11.8)	70 (11.3)	28 (13.1)	0.46
**Coronary angiography, n (%)**	502 (60.3)	488 (78.7)	14 (6.6)	<0.0001
**Total PCI, n (%)**	399 (47.9)	394 (63.5)	5 (2.3)	<0.0001
**STEMI and reperfusion therapy (n = 506 patients), n (%)**	––––	187 (37.0)	––––	––––
**Primary PCI, n (%)**	––––	175 (34.6)	––––	––––

IABP, intra-aortic balloon pump; PCI, percutaneous coronary intervention.

## Predictors of in-hospital mortality

**Figure 4:**
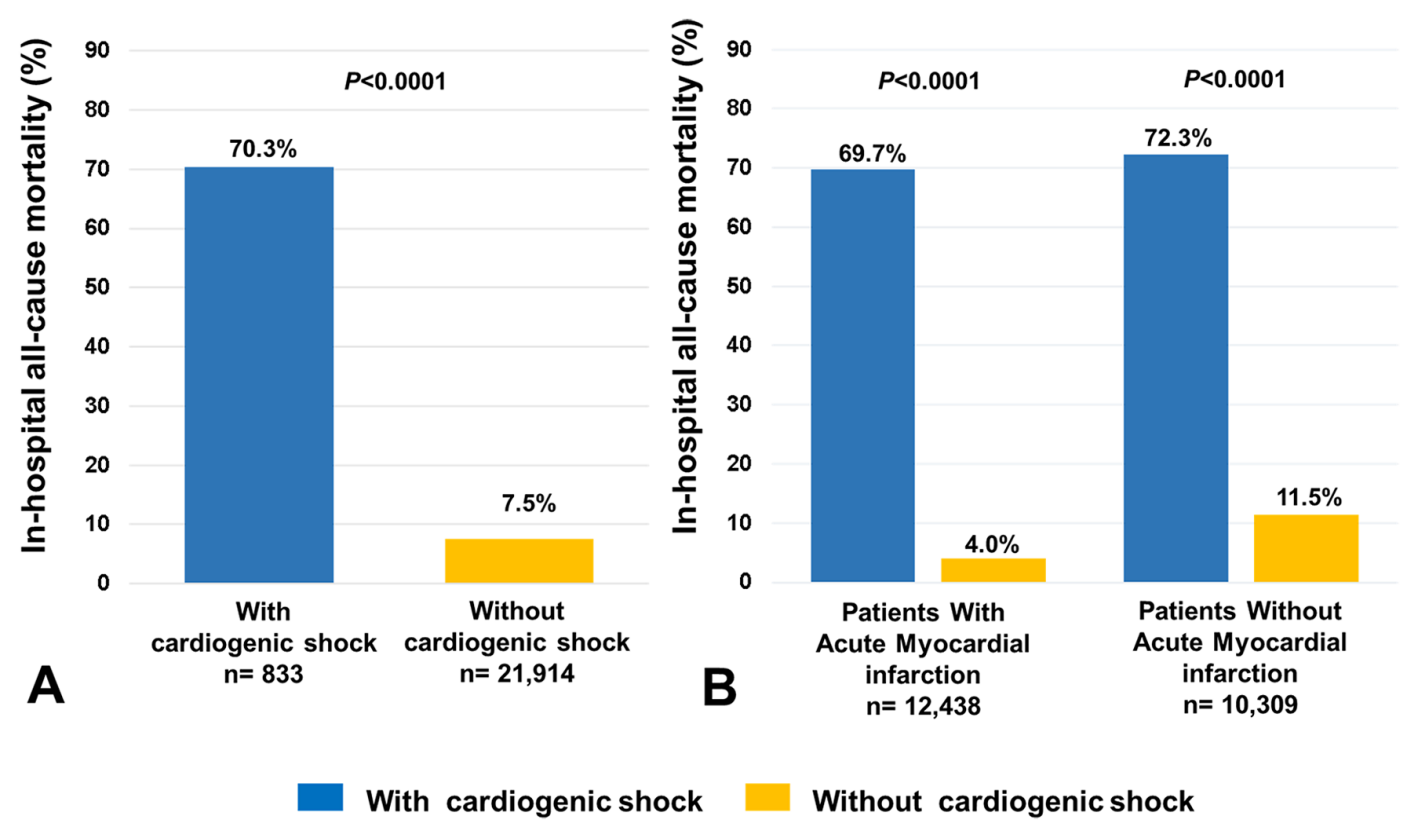
**A** Overall in-hospital all-cause mortality rates of the 22,747 CCU admissions with and without cardiogenic shock (CS). **B.** In-hospital all-cause mortality rates among patients with CS in groups with and without acute myocardial infarction (AMI).

In the whole cohort, in-hospital all-cause mortality in patients with CS over the entire study period was 70.3% (n = 586), compared with 7.5% of those who did not develop CS (P < 0.0001, Figure 4-A). In both the AMI and non-AMI patient groups, the unadjusted all-cause mortality rate in hospital was higher in patients with CS compared with patients without CS (AMI–CS, n = 432, 69.7% vs non-AMI–CS; 154, 72.3%, P = 0.47; Figure 4-B).

Using the patient group without CS as a reference in a Cox proportional hazards model adjusted for age and gender, we found that patients in the AMI–CS group had a 9.91-fold increased risk of in-hospital mortality (HR 9.91, 95% CI 8.90–12.36; P < 0.0001). Patients in the non-AMI–CS group had a 10.47-fold increased risk of in-hospital mortality (HR 10.47, 95% CI 8.87–12.77; P < 0.0001) compared with those who did not develop CS.

Among the 586 patient deaths, 81.1% (n = 475) were of CV causes and 18.9% (n = 111) were of other causes. CV death was more frequent in patients with AMI–CS than in patients with non-AMI–CS (n = 369; 85.4% vs n = 106; 68.8%, respectively). In contrast, the non-CV death rate was 14.6% (n = 63) in the AMI–CS group versus 31.2% (n = 48) in the non-AMI–CS group.

Adjusted multivariate Cox proportional hazards regression models were generated with all statistically significant univariate predictors of in-hospital mortality, listed in Tables 5 and 6. In the AMI–CS patients, independent predictors of in-hospital all-cause mortality were renal dysfunction (creatinine depuration <30 mL/min [HR 1.94, 95% CI 1.34–2.80; P < 0.0001]; creatinine depuration 30–59 mL/min [HR 1.36, 95% CI 1.01–1.86; P < 0.05]), LVEF (per 5% decrease; HR 1.13, 95% CI 1.06–1.20; P < 0.0001), diabetes (HR 1.31, 95% CI 1.01–1.69; P = 0.04) and age (per 10-y group, HR 1.19, 95% CI 1.05–1.35; P = 0.006). Furthermore, the absence of PCI (HR 1.44, 95% CI 1.10–1.90; P = 0.008) and the need for mechanical ventilation (HR 1.49, 95% CI 1.10–2.01, P = 0.01) were also independent predictors of in-hospital all-cause mortality. The type of acute coronary syndrome, as well as the use of an intra-aortic balloon pump were not associated with an increased risk of mortality (Table 7). On the other hand, in patients with non-AMI CS, the independent predictors were only the LVEF (per 5% decrease; HR 1.05, 5% CI 1.04–1.10; P = 0.05) and blood lactate levels >2.0 mmol/L at the time of admission (HR 1.66, 95% CI 1.13–2.45; P = 0.01).

**Table 5:** Univariable analysis for the prediction of in-hospital all-cause mortality in patients with cardiogenic shock associated with acute myocardial infarction (AMI-CS).

	Hazard ratio	95% Confidence Interval	P Value

**Gender (Female)**	1.38	1.12 to 1.72	0.003
**Age (per 10years)**	1.21	1.12 to 1.32	<0.0001
**Diabetes**	1.47	1.21 to 1.78	<0.0001
**STEMI**	1.10	0.86 to 1.41	0.42
**NSTEMI**	0.90	0.70 to 1.15	0.42
**Cardiogenic shock to admission**	1.34	1.11 to 1.62	0.002
**LVEF (per 5% decrease)**	1.10	1.04 to 1.15	<0.0001
**Systolic blood pressure (per 10 mmHg decrease)**	1.05	1.02 to 1.09	0.002
**Diastolic blood pressure (per 10 mmHg decrease)**	1.11	1.05 to 1.17	<0.0001
**Mean blood pressure (per 10 mmHg decrease)**	1.09	1.04 to 1.15	<0.0001
**Renal dysfunction**			
**Creatinine depuration ≥60 mL/min**		Reference group	
**Creatinine depuration 30–59 mL/min**	1.82	1.44 to 2.96	<0.0001
**Creatinine depuration <30 mL/min**	2.70	2.09 to 3.48	<0.0001
**Blood Lactate, >2.0 mmol/L**	1.32	1.09 to 1.60	0.004
**NT-proBNP (pg/mL)**	1.0	1.0 to 1.0	0.005
**Absence percutaneous coronary intervention**	1.75	1.44 to 2.12	<0.0001
**Intra-aortic balloon pump**	0.86	0.71 to 1.04	0.13
**Mechanical ventilation**	1.27	1.02 to 1.58	0.03

MI, myocardial infarction; CABG, coronary artery bypass grafting; PCI, percutaneous coronary intervention; STEMI, ST-segment-elevation myocardial infarction; NSTEMI, non-ST-segment elevation Myocardial Infarction; LVEF, left ventricular ejection fraction; hs-CRP, high-sensitivity C-reactive protein; cTnI, cardiac troponin I; NT-proBNP, N-terminal pro-brain natriuretic peptide.

**Table 6:** Univariable analysis for the prediction of in-hospital all-cause mortality in patients with cardiogenic shock no associated with acute myocardial infarction (non-AMI-CS).

	Hazard ratio	95% Confidence Interval	P Value

**Cardiogenic shock to admission**	1.46	1.02 to 2.10	0.03
**LVEF (per 5% decrease)**	1.05	1.00 to 1.10	0.05
**Systolic blood pressure (per 10 mmHg decrease)**	1.08	1.00 to 1.17	0.02
**Diastolic blood pressure (per 10 mmHg decrease)**	1.13	1.00 to 1.27	0.03
**Mean blood pressure (per 10 mmHg decrease)**	1.11	1.01 to 1.21	0.02
**Blood Lactate, >2.0 mmol/L**	1.75	1.19 to 2.56	0.004
**Valvular surgery**	0.04	0.00 to 0.89	0.04

MI, myocardial infarction; CABG, coronary artery bypass grafting; PCI, percutaneous coronary intervention; STEMI, ST-segment-elevation myocardial infarction; NSTEMI, non-ST-segment elevation Myocardial Infarction; LVEF, left ventricular ejection fraction; hs-CRP, high-sensitivity C-reactive protein; cTnI, cardiac troponin I; NT-proBNP, N-terminal pro-brain natriuretic peptide.

**Table 7:** Independent predictors of in-hospital all-cause mortality in patients cardiogenic shock associated with acute myocardial infarction (AMI-CS).

	Hazard ratio	95% Confidence Interval	P Value

**Age (per 10years)**	1.19	1.05 to 1.35	0.006
**Diabetes**	1.31	1.01 to 1.69	0.04
**LVEF (per 5% decrease)**	1.13	1.06 to 1.20	<0.0001
**Creatinine depuration ≥60 mL/min**		Reference group	
**Creatinine depuration 30–59 mL/min**	1.36	1.01 to 1.86	0.05
**Creatinine depuration <30 mL/min**	1.94	1.34 to 2.80	<0.0001
**Absence PCI**	1.44	1.10 to 1.90	0.008
**Mechanical ventilation**	1.49	1.10 to 2.01	0.01

PCI, percutaneous coronary intervention; LVEF, left ventricular ejection fraction.

In addition, patients with AMI-CS were more likely to develop other important clinical complications in comparison to non-AMI-CS patients; such as reinfarction (6% vs 0%; P < 0.0001), ventricular arrhythmias (39.5% vs 28.2%; P = 0.003), third-degree atrioventricular blocks (11.1% vs 1.4%; P < 0.0001), major bleeding (5.8% vs 0.5%; P = 0.001), and nosocomial pneumonia (17.6% vs 7.5%; P < 0.0001) (Table 8).

**Table 8:** In-hospital adverse events.

	Overall (n = 833)	AMI-CS (n = 620)	Non-AMI-CS (n = 213)	*P*- Value

**Reinfarction/infarction, %**	4.4	6.0	0.0	<0.0001
**Ventricular arrhythmias, %**	36.6	39.5	28.2	0.003
**Atrial fibrillation, %**	7.8	7.9	7.5	0.85
**Third-degree atrioventricular block, %**	8.6	11.1	1.4	<0.0001
**Stroke (%)(any type)**	2.8	3.1	1.9	0.36
**Major bleeding (%)**	4.4	5.8	0.5	0.001
**Nosocomial pneumonia, %**	15.0	17.6	7.5	<0.0001

AMI-CS, Cardiogenic Shock caused by Acute myocardial infarction; non-AMI-CS, Cardiogenic shock of non-ischemic etiology.

## Discussion

Here we describe the prevalence, temporal trends, characteristics and in-hospital outcomes of consecutive patients with AMI-CS and non-AMI-CS admitted to the CCU of a tertiary medical center specializing in cardiovascular diseases in Mexico, a middle income country in Latin America. Our major findings were an upward trend in prevalence rates over time in the group of patients with non-AMI-CS and the prevalence remained unchanged over time in AMI-CS. In comparison to prior publications we found significant differences in demographic characteristics, different treatment approaches and very poor prognosis despite young age when compared with similar studies conducted in high-income countries in Europe and the USA.

However, the overall prevalence rates for CS associated with AMI were similar to those reported in the large registries in Europe and the USA. Information concerning the incidence of CS comes mainly from large registries of patients with AMI, showing rates of 3–10%, depending in part on the prevailing definitions of CS and the characteristics of the population studied [5,10,14,15]. Nevertheless, few studies have evaluated the full spectrum of CS (AMI–CS and non-AMI–CS) in contemporary CCUs in Latin America. Our findings are similar to those reported in previous studies. For example, Harjola et al. [4] in a contemporary registry reported 219 patients with CS; 81% (n = 177) of the patients had ACS while other non-ACS etiologies represented 19% (n = 42). In our population of nonselected patients with CS, AMI was the most common cause in all cases (74.4%); other causes (non-AMI–CS) were associated with the remaining quarter. Likewise, the overall prevalence of CS in patients with AMI (5%) observed here falls within the range reported in the literature and shows the highest frequency in patients with STEMI compared with those with NSTEMI [5,10,14,15]. Previous studies have established that older patients and the presence of cardiac comorbidities are risk factors for developing CS after AMI [1,16]. However, the findings of the current study show differences in demographic data, clinical characteristics and co-morbidities compared with the large registries from the USA and Europe [5,10]. In our study population, the patients with AMI–CS were younger (by approximately one decade) with a higher prevalence of diabetes mellitus. Importantly, the prevalence rates of hypertension and previous myocardial infarction were similar. Another important fact was the patients delayed presentation at hospital admission after the starting of AMI symptoms (median 17.2 hours), which is common in low- and middle-income countries bringing out a much higher rate of patients that did not receive reperfusion therapy [17,18].

On the other hand, in the non-AMI–CS group of patients, a history of chronic heart failure was more frequent than that reported in other studies (75% vs. 62%) [19].

Previous studies have shown that a minority of patients with AMI develop CS before hospital admission, whereas the majority developed this complication during acute hospitalization [20,21,22]. In contrast, in our study population more than half of the CS patients presented with CS on admission (AMI–CS, 54.2% and non-AMI–CS 74.6%). These findings are consistent with the most recent study where approximately half of the patients presented with CS at the time of admission [10].

Cardiogenic shock is thought to represent the most severe form of acute heart failure in multiple pathological conditions, and identification of the underlying cause can permit the initiation of specific pharmacological or mechanical therapies. In our study, among all patients with AMI–CS, left ventricular failure was the most common cause (71.3%), right heart failure in 13.5% and mechanical complications such as acute severe mitral regurgitation, ventricular septal rupture, free wall rupture and cardiac tamponade was seen in 15% of cases. These findings are in accordance with those reported from the SHOCK trial; left ventricular failure in 80% of cases and mechanical complications in the remaining 20% [2].

At present, there is a scarcity of information on nonischemic etiologies for CS. Many affected patients have acute worsening of their chronic disease or less common acute causes such as myocarditis, stress-induced cardiomyopathy, peripartum cardiomyopathy or prosthetic dysfunction [12]. Comparing our findings with other studies, we found that the proportion of patients with CS of nonischemic etiology was greater than that reported elsewhere (25% vs 19%); the etiologies in 91.6% of patients with non-AMI–CS were valvular heart disease and cardiomyopathies. However, within these two large groups, the etiology was very heterogeneous (Figure 3) [4,19]. One possible explanation for this finding might be the epidemiological characteristics of our study population and the type of hospital where these data were collected.

One interesting finding was the high rate of in-hospital mortality among the patients with both AMI–CS (69%) and non-AMI–CS (70%), contrasting with the published rates of 40–60% for AMI–CS [7,9,13], and 24–36% for non-AMI–CS [4,19]. In AMI–CS patients, multivariate analyses showed that factors associated with in-hospital mortality were consistent with those reported previously [23,24,25], namely renal dysfunction, diabetes, older age, and severely depressed LVEF along with the absence of PCI and increased use of mechanical ventilation. However, treating patients with CS in tertiary centers with the availability of primary PCI and access to mechanical circulatory support (MCS) might potentially increase the survival of these patients [26,27].

The prognosis of patients with AMI complicated by CS has improved over the past decade mainly thanks to early revascularization [9,10]. The higher rates of in-hospital mortality found in our population could be attributed, on one hand in part, to the advanced hemodynamic and metabolic shock state of patients at admission (lactic acidosis, renal impairment, and liver injury), on the other by the decrease amount of an early revascularization. In our study population, primary PCI was only performed in a third of patients with STEMI, which suggests that most patients with STEMI delayed their attendance at the hospital. On the other hand, in our analysis the use of intra-aortic balloon pump (IABP) was not a predictor of survival, a finding consistent with a previous study [8]. While several reports have shown that IABP is now less often used, application of other MCS has increased both in Europe and in the USA [7,28,29]. In our CCU, IABP is the most widely used MCS device (47.4%) because other advanced forms of MCS are not yet available to us. It seems possible that our poor mortality results could have been better if we had access to these advanced forms of MCS [30].

In the group of patients with non-AMI–CS, we could identify only two predictors, depressed LVEF and high blood lactate levels, associated with high mortality. However, the majority of these patients had history of previous heart failure (75%), added to this the non-CV death was presented in one-third of them, so this might indicate indirectly, that this was a high-risk patient cohort with end-stage heart failure caused by advanced underlying heart diseases.

Recently, The Heart Failure Association of the European Society of Cardiology has suggested that, despite advances in therapy, CS remains the most common cause of in-hospital death after AMI and is a major cause of death in young patients with other potentially reversible underlying cardiac pathologies. According to the Heart Failure Association, CS management should consider appropriate organization of the health-care services, and therapies must be given to appropriately selected patients in a timely manner, while avoiding iatrogenic harm. This association also suggested that further research is needed for the identification of the new pathophysiological targets, and high-quality translational research should facilitate incorporation of more targeted interventions in clinical research protocols, aimed to improve individual patient outcomes [31].

Findings from this study in a middle-income country provide more information of the clinical characteristics, etiologies and outcome that differ significantly from that reported in high-income countries. Better understanding of these findings may suggest approaches to improve CS outcomes in developing countries.

## Study limitations

Our study had certain limitations. First, there was the inherent limitation of a retrospective analysis and the fact that it reflected the experiences of a single tertiary center specializing in cardiovascular diseases. Second, these data do not provide an overview of patients with CS treated throughout Latin America. As our study was carried out in a tertiary referral hospital in Mexico City with well-defined demographic characteristics, the data are not representative of the whole country.

Finally, the database did not allow us to determine the time between the onset of CS and the time of hospital admission for the group of patients with CS at admission, as well as the time elapsed from admission to the development of CS in those patients with late CS.

## Conclusions

Hospitalized patients with CS include patients with AMI–CS and those with non-AMI–CS, and they can have an entirely different pathophysiology. As a result, we recommend that it is highly important to identify the cause of the underlying CV disease in order to allow the initiation of specific therapies. Furthermore, by including CS across all etiologies in our study, we have provided additional information on the high morbidity and mortality associated with CS in the presence and in absence of AMI in a tertiary reference center specialized in CV diseases in Mexico, a middle-income country in Latin America. These findings highlight the need for continued research and the importance of further studies in low- and middle-income countries to evaluate the efficacy of existing strategies for the prevention and treatment of CS in hospitalized patients with CV disease, and the development of protocols to ensure the optimal use of effective and up-to-date treatment strategies.

In developing countries such as Mexico, unfavorable social circumstances, along with inadequate and inefficient public spending on health care, can present considerable barriers to improve outcomes in patients with CS. There is a need to develop more efficient strategies to identify in a timely manner patients at high risk for developing CS or with impending CS who are likely to need a higher level of care, and discussions about possible transfer should occur early in the clinical course. Such strategies should be based on multidisciplinary models involving CS teams, structured referral schemes and standardized cardiovascular intensive care units. Mexican researchers in this field are looking forward to regionalizing networks dedicated to CS and starting up collaboration programs between non-PCI capable centers and specialized tertiary hospitals with multidisciplinary CS teams, according to the ‘hub and spoke’ model, in order to coordinate the most appropriate and time-effective therapeutic strategy.
